# Bone-induced streak artifact suppression in sparse-view CT image reconstruction

**DOI:** 10.1186/1475-925X-11-44

**Published:** 2012-08-02

**Authors:** Seung Oh Jin, Jae Gon Kim, Soo Yeol Lee, Oh-Kyong Kwon

**Affiliations:** 1 Korea Electrotechnology Research Institute, Seoul, Korea; 2 Department of Biomedical Engineering, Kyung Hee University, Seoul, Korea; 3 Department of Electronic Engineering, Hanyang University, Seoul, Korea

**Keywords:** Streak artifact, Sparse-view CT, Compressed sensing, ART, Total variation

## Abstract

**Background:**

In sparse-view CT imaging, strong streak artifacts may appear around bony structures and they often compromise the image readability. Compressed sensing (CS) or total variation (TV) minimization-based image reconstruction method has reduced the streak artifacts to a great extent, but, sparse-view CT imaging still suffers from residual streak artifacts. We introduce a new bone-induced streak artifact reduction method in the CS-based image reconstruction.

**Methods:**

We firstly identify the high-intensity bony regions from the image reconstructed by the filtered backprojection (FBP) method, and we calculate the sinogram stemming from the bony regions only. Then, we subtract the calculated sinogram, which stands for the bony regions, from the measured sinogram before performing the CS-based image reconstruction. The image reconstructed from the subtracted sinogram will stand for the soft tissues with little streak artifacts on it. To restore the original image intensity in the bony regions, we add the bony region image, which has been identified from the FBP image, to the soft tissue image to form a combined image. Then, we perform the CS-based image reconstruction again on the measured sinogram using the combined image as the initial condition of the iteration. For experimental validation of the proposed method, we take images of a contrast phantom and a rat using a micro-CT and we evaluate the reconstructed images based on two figures of merit, relative mean square error and total variation caused by the streak artifacts.

**Results:**

The images reconstructed by the proposed method have been found to have smaller streak artifacts than the ones reconstructed by the original CS-based method when visually inspected. The quantitative image evaluation studies have also shown that the proposed method outperforms the conventional CS-based method.

**Conclusions:**

The proposed method can effectively suppress streak artifacts stemming from bony structures in sparse-view CT imaging.

## Background

Sparse-view CT is of great importance in clinical imaging for its potential to reduce the x-ray dose to the human subject and the scan time [1-3]. In sparse-view CT, less number of projection views than is required to satisfy the Nyquist sampling theorem is employed. Conventional filtered backprojection (FBP) based image reconstruction methods gives severe streak artifacts, sort of aliasing artifacts, in the images, which would hamper clinical utility of the sparse-view CT. Bony structures makes strongest streak artifacts, and physiological motions of the human subject, beam hardening, and photon starvation also make streak artifacts [4-7]. In sparse-view CT image reconstruction, iterative image reconstruction methods are usually employed since they outperform the conventional FBP methods in terms of signal-to-noise ratio (SNR) and streak artifacts [8-10]. Recent developments of compressed sensing (CS) or total variation (TV) minimization-based image reconstruction methods have reduced streak artifacts to the extent that sparse-view CT would be a plausible imaging modality for some clinical applications [11,12]. Imaging guided radiation therapy (IGRT) using a cone-beam CT (CBCT) is one of the applications of great interests [13]. It is now widely recognized that the CS-based image reconstruction can suppress streak artifacts to the unnoticeable level in the case of simple-structured-phantom imaging with the number of views as small as several tens [14-19]. But, in human imaging in which sparsity of the images is much lower than in the phantom imaging case, the CS-based image reconstruction methods often fail in suppressing streak artifacts. There have been a few reports on streak artifact suppression techniques in the CS-based image reconstruction. Leng et al. introduced a method to suppress respiration-induced streak artifacts in four-dimensional CBCT [20]. They used a full-view image as a prior to suppress the streak artifacts in each respiratory phase image. They also proposed a method that a full-view image be used as a prior for the constraint in the CS-based image reconstruction from highly sparse-view projection data [15].

In this paper, we introduce a new sparse-view image reconstruction method to further reduce streak artifacts stemming from high-intensity objects like bony structures or metal implants. We incorporate bone segmentation into the CS-based image reconstruction to prevent streak artifact formation in the soft tissue regions. We have verified the proposed method using the projection data obtained from micro-CT scanning of a contrast phantom and a laboratory rat.

## Methods

### ART and CS

We use the algebraic reconstruction technique (ART) and CS as a platform for the sparse-view image reconstruction. ART is a minimum mean square error (MMSE) solver to find the image **f** that best matches the measured projection data **g**:

(1) Af=g

where **A** is the system matrix describing the forward projection in the CT scan [21]. In ART, the above equation is solved in an iterative way that the difference between the projection data measured in the real scan and the projection data calculated from the estimated image is back-projected on to the image estimated at the previous iteration step. ART is known to have better performance than FBP in suppressing streak artifacts in sparse-view imaging. Many variants of ART with different iteration schemes have been proposed to improve the image quality and to reduce the computation time [22-25]. In this study, we use the ordered-subset simultaneous algebraic reconstruction technique (OS-SART) [24] for an ART solver.

The CS-based image reconstruction methods solve the following constrained optimization problem which has constraints of data fidelity and pixel positivity [26]:

(2) $$\underset{f}{\text{arg min}}{\left|\Psi f\right|}_{l_{1}}\;s.t.\;Af=g\text{,}\;f\geq 0$$

where Ψ is a sparsifying transform operator, and ${\left|z\right|}_{l_{1}}=\sum_{i=1}^{N}\left|z_{i}\right|$ is the *l*_1_ norm of an *N*-dimensional vector **z**. In this study, we use the discrete gradient transform for the sparsifying transform which has been widely used in the CS-based image reconstruction [14]:

(3) $$\Psi f=\sqrt{{\left[f\left(i+1,j\right)-f\left(i,j\right)\right]}^{2}+{\left[f\left(i,j+1\right)-f\left(i,j\right)\right]}^{2}}$$

where *i* and *j* are the pixel indices in the *x*- and *y*-directions, respectively, and *f* is the 2D matrix form of **f**. The discrete gradient transform is often denoted as TV. We implement the CS-based image reconstruction algorithm using the OS-SART to enforce the data fidelity and the steepest descent method to minimize the TV in an alternating manner in the iteration. We summarize the algorithm for the CS-based image reconstruction in the following pseudo-code [19,27].

(4) $$function\;{\text{CS}}_{\text{TV}}\left(g\text{,}\beta \text{,}\beta_{\text{red}}\text{K}\text{,}f^{\text{init}}\right)$$

1. **f**^0^ : = **f**^init^;

2. for k = 1: 1: K(main loop)

3. update f^k^ by OS-SART from the projection data **g**;

4. for *l =* 1: 1: 10 (TV minimization loop)

5. compute the steepest decent direction **d** of TV;

6. $\text{ρ}=\max\left(\left|{\text{f}}^{k}\right|\right)÷\max\left(\left|\text{d}\right|\right)$;

7. $f^{k}=f^{k}-\text{β}\times \text{ρ}\times d$;

8. end

9. $\text{β}=\text{β}\times {\text{β}}_{\text{red}}$;

10. end

11. return f^k^

The TV-minimization step has two control parameters, the maximum step size β in the steepest descent search, the reduction factor β_red_ of the maximum step size after each iteration of the main loop. It is commonly known that the large step size of the steepest descent makes the image look smooth, and the small one makes the image look sharp [13,14]. In this study, we empirically choose β and β_red_ considering that too large β makes the image weak-contrasted whilst too small β makes the image very similar to the one reconstructed by ART [14].

### Streak-artifact-suppressed CS image reconstruction (SAS-CS)

**Figure 1:**
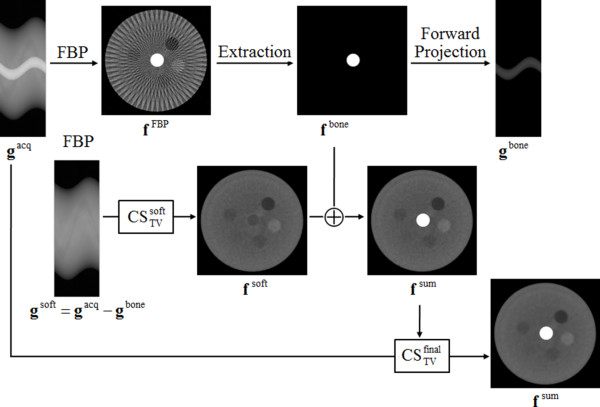
Image reconstruction procedure to suppress the streak artifacts.

To reduce streak artifacts stemming from high-intensity structures like bones or metal implants, we combine CS-based image reconstruction approaches with the conventional FBP. Figure 1 shows the basic idea of the proposed method.

Step 1 : $f^{\text{FBP}}=\text{FBP}\left(g^{\text{acq}}\right)$

To identify the high-intensity region that makes strong streak artifacts, we first reconstruct an image using FBP, **f**^FBP^, from the acquired sinogram **g**^acq^. The resulting image may have streak artifacts around the high-intensity structures.

Step 2 : Extracting **f**^bone^ from **f**^FBP^

From **f**^FBP^**,** we extract the high-intensity region, denoted as **f**^bone^, by applying a thresholding technique. We manually choose the global threshold *T*^bone^ by visual inspection of the image histogram.

Step 3 : Computing **g**^bone^ by forward projecting **f**^bone^

From **f**^bone^, we compute forward projection of the high-intensity region to make the sinogram data **g**^bone^ that accounts for the high-intensity region only.

Step 4 : $g^{\text{soft}}=g^{\text{acq}}-g^{\text{bone}}$

We subtract **g**^bone^ from the measured sinogram, **g**^acq^, to exclude the components stemming from the high-intensity region.

Step 5 : $f^{\text{soft}}={\text{CS}}_{\text{TV}}\left(g^{\text{soft}},\text{β}=0.0060,{\text{β}}_{\text{red}}=0.98,\text{K}=30,f^{\text{init}}=0\right)$]

We use the subtracted sinogram **g**^soft^, which account for the soft tissues only, for reconstruction of soft tissue images using the CS-based method. In this step of CS-based image reconstruction, we use a uniform image of zeroes as an initial guess of the CS-based image reconstruction.

Step 6 : $f^{\text{sum}}=f^{\text{bone}}+f^{\text{soft}}$

After reconstructing the soft tissue image **f**^soft^ via CS, we add the high-intensity region image **f**^bone^, which has been reconstructed by FBP, to the soft tissue image to get the composite image **f**^sum^.

Step 7 : $f^{\text{final}}={\text{CS}}_{\text{TV}}\left(g^{\text{acq}},\text{β}=0.0033,{\text{β}}_{\text{red}}=0.98,\text{K}=30,f^{\text{init}}=f^{\text{sum}}\right)$.

To further refine the CT image, we perform the CS-based iterations again on the original sinogram with the initial guess of the CT image set to f^sum^obtained at the last step.

The CS-based image reconstruction in step 5 and 7 solves the constrained minimization problem defined in Eq. (2). Step 5 needs two inputs and three control parameters. The two inputs are the soft tissue sinogram, g^soft^, and the initial guess of the reconstructed image which is all zeroed. The three control parameters are β and β_red_ defined in the previous section, and K the maximum number of iterations of the main loop. In step 7, we perform the CS-based image reconstruction again using the same procedure as in step 5, but with the data inputs of g^acq^ and f^sum^ which has been obtained in step 6.

### Data acquisition

We have performed all the CT scans using the lab-built micro CT system described in our previous work [28]. The micro CT system consists of a micro-focus x-ray source, a rotating object holder, a CMOS flat-panel detector. The micro-focus x-ray source (L8121-01, Hamamatsu, Japan) has a fixed tungsten anode having an angle of 25° against the electron beam and a 200 μm-thick beryllium exit window. The emitted x-ray beam has a span angle of 43°. The source has a variable focal spot size from 5 μm to 50 μm depending on the applied tube power. We have operated the micro-focus x-ray source in a continuous mode with a 1 mm-thick Al filter. We have used a commercially available flat-panel detector (C7942, Hamamatsu, Japan) as a 2D digital x-ray imager in the micro-CT system. The flat-panel detector consists of a 2240 × 2240 active matrix of transistors and photodiodes with a pixel pitch of 50 μm, and a CsI:Tl scintillator.

**Figure 2:**
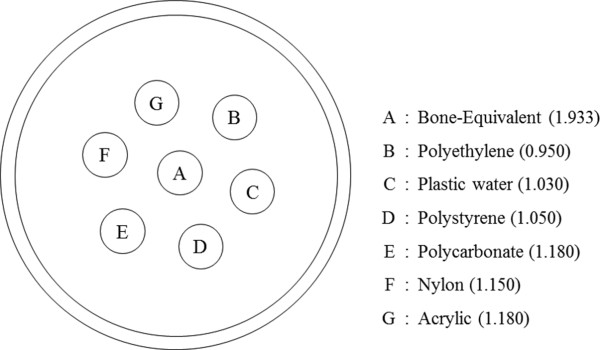
**A schematic diagram of the contrast phantom.** Physical densities of the inserts (g/cm^3^) are shown in the parentheses.

To validate the proposed method, we have performed CT scans of a contrast phantom and a sacrificed adult rat using the micro-CT. The contrast phantom consists of seven inserts six of which have physical densities similar to that of water and the rest of which has the bone-equivalent physical density. Figure 2 shows a schematic diagram of the contrast phantom along with the physical densities of the inserts. The seven inserts of 5 mm diameter were in a water bath made of an acryl cylinder of 40 mm diameter. We have made the inserts using the commercial electron density phantoms (Model 76-430, Nuclear Associates, NY, USA). We applied tube voltage and current of 40 kVp and 0.5 mA for the contrast phantom imaging, and 65 kVp and 0.34 mA for the rat imaging, respectively. To get reference images, we performed full-view scans with the number of views of 900 over 360 degrees. To get sparse-view projection data, we decimated the full-view projection data in the view direction.

### Image quality evaluation

In order to evaluate the final image quality particularly in terms of streak artifact formation, we use two metrics, one for the total variations stemming from streak artifacts and the other for the relative errors of the final image with respect to the reference image. We define the normalized streak indicator (*SI*) using the total variation of the difference image [12] :

(5) $$SI=\frac{\text{TV}\left(f-f^{\text{ref}}\right)}{\text{TV}\left(f^{\text{FBP}}-f^{\text{ref}}\right)}$$

where **f** is the sparse-view image reconstructed by the proposed method, **f**^ref^ the reference full-view image reconstructed by FBP, and **f**^FBP^ the sparse-view image reconstructed by FBP, respectively. We calculate TV using Eq. (3). For the reference images, we use the 900-view images reconstucted by FBP which have little streak artifacts.

To evaluate reconstruction errors as compared to the reference image, we use the relative root mean square error (RRME) defined by [12]:

(6) $$RRME=\sqrt{\frac{\sum_{i,j}{\left(f\left(i,j\right)-f^{\mathrm{ref}}\left(i,j\right)\right)}^{2}}{\sum_{i,j}f^{\text{ref}}{\left(i,j\right)}^{2}}}\text{,}$$

where *f* is the matrix form of the image vector **f**.

## Results

**Figure 3:**
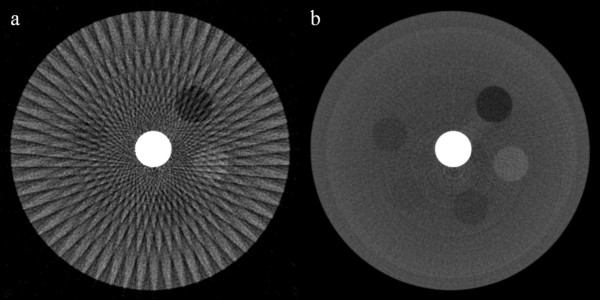
**Contrast phantom images reconstructed by FBP.** The images have been reconstructed from **a** 60-view and **b** 900-view projection data. The images are normalized to 1.0. The display window is [0.05 0.45].

**Figure 4:**
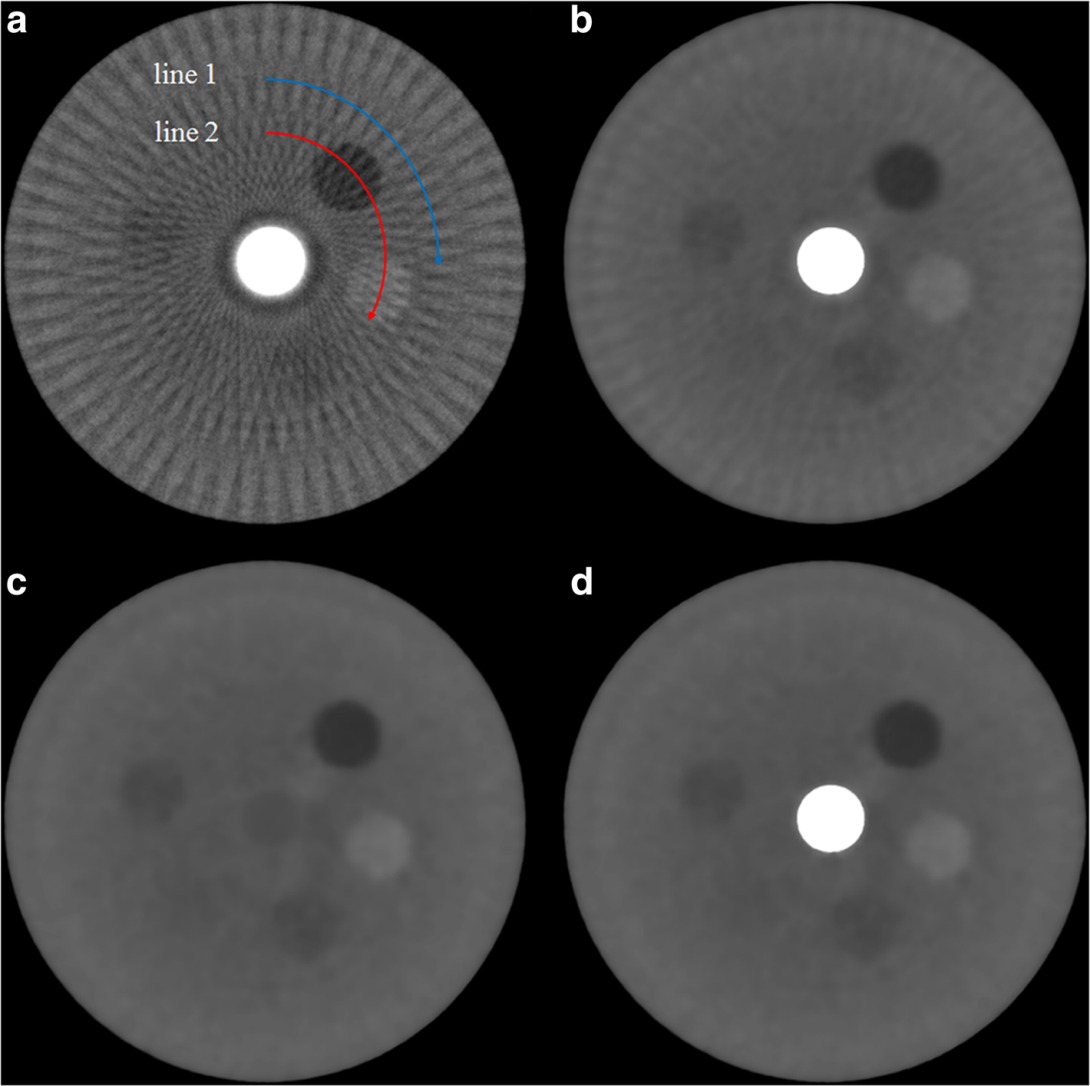
**Contrast phantom images reconstructed by the iterative methods.** Three iterative reconstruction methods, **a** ART, **b** CS and **d** SAS-CS, have been used. The soft tissue image **c** has been reconstructed in step 5 of SAS-CS. The images are normalized to 1.0. The display window is [0.05 0.45].

**Figure 5:**
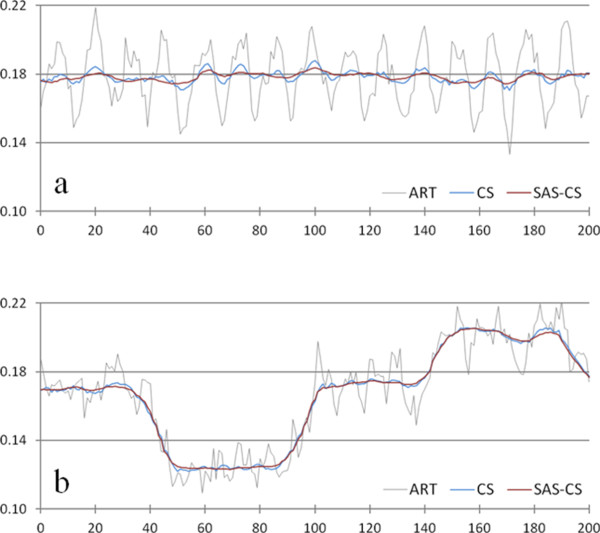
**Pixel-intensity profiles of the contrast phantom images along the lines shown in Figure**4**a.** Three iterative reconstruction methods, ART, CS, and SAS-CS, are compared for the 60-view imaging. The pixel intensity profiles **a** along the line 1 and **b** along the line 2 in Figure 4a are shown.

We have first reconstructed contrast phantom images the size of 512 × 512 using FBP from the 60-, and 900-view projection data acquired from the micro-CT scan. The images shown in Figure 3 have the pixel size of 85 × 85 μm^2^. As can be noticed from Figure 3b, the 900-view image to be used as a reference image shows little streak artifacts whilst the 60-view image shows strong streak artifacts stemming from the high-intensity insert placed at the center of the phantom. We have reconstructed the contrast phantom images using the three iterative reconstruction methods, ART (Figure 4a), CS (Figure 4b) and SAS-CS (Figure 4d). Figure 4c shows the soft-intensity image calculated in step 5 in SAS-CS. From the images, we can see that the ART image has strongest streak artifacts. In the CS image, the streak artifact has been remarkably reduced, but we can still see the residual streak artifacts. The SAS-CS image shows least level of steak artifacts among the three images. Figure 5 shows pixel-intensity profiles along the two lines shown in Figure 4a. Along the line 1 which runs the uniform background region, SAS-CS shows least level of fluctuation demonstrating its performance of streak-artifact suppression. Along the line 2 which runs over the inserts, CS and SAS-CS show similar level of fluctuations. Table 1 summarizes the RRMEs and SIs of the images reconstructed by the aforementioned three methods with respect to the reference image. Due to the strong streak artifacts, the ART image shows the biggest RRMEs whilst the SAS-CS image shows the least RRMEs. In terms of the normalized SI, the ART image also shows the worst performance and the SAS-CS image shows the best performance.

**Table 1:** Means and standard deviations, RRMEs and SIs in the contrast phantom images

	**Contrast Phantom Case**		
**Reconstruction Methods**	**Mean values ± standard deviations**	**RRME**	**SI**
ART	0.1787 ± 0.0172	0.0095	0.4471
CS	0.1784 ± 0.0033	0.0032	0.3014
SAS-CS	0.1783 ± 0.0022	0.0027	0.2966

Three iterative reconstruction methods, ART, CS, and SAS-CS, are compared for the 60-view imaging. Mean values and standard deviations have been calculated from the pixel-intensity profiles in Figure 5a.

**Figure 6:**
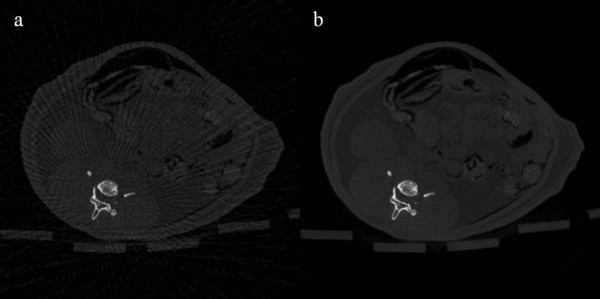
**Rat abdomen images reconstructed by FBP.** The images have been reconstructed from **a** 100-view and **b** 900-view projection data. The images are normalized to 1.0. The display window is [0.01 0.50]

**Figure 7:**
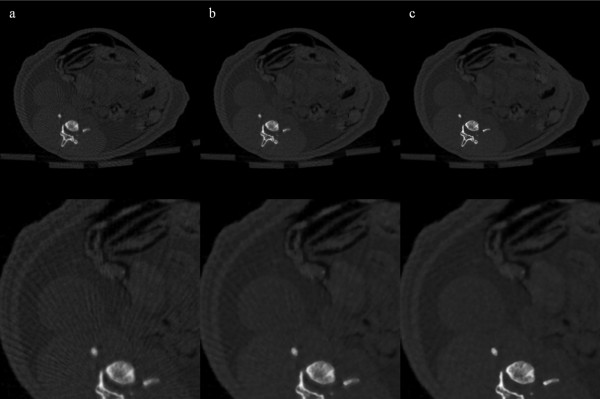
**Rat abdomen images reconstructed by the iterative methods.** Three iterative reconstruction methods, ART(**a**), CS(**b**) and SAS-CS(**c**), have been used for 100-view imaging. The images are normalized to 1.0. The display window is [0.01 0.50]. Magnified images are shown in the bottom row.

**Figure 8:**
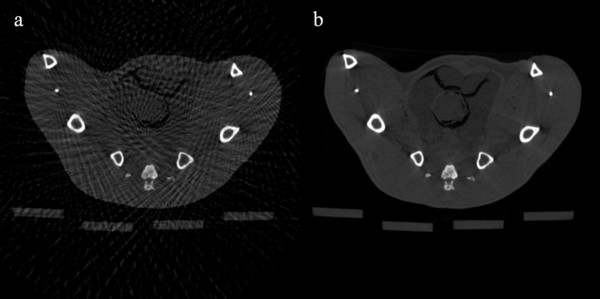
**Rat pelvic floor images reconstructed by FBP.** The images have been reconstructed from **a** 100-view, **b** 900-view projection data. The images are normalized to 1.0. The display window is [0.01 0.55].

**Figure 9:**
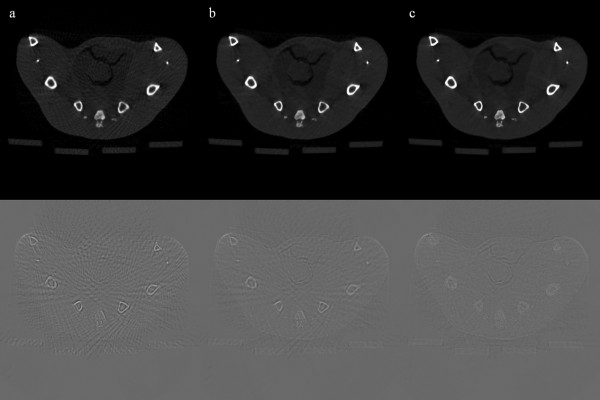
**Rat pelvic floor images reconstructed by the iterative methods.** Three iterative reconstruction methods, ART (**a**), CS (**b**) and SAS-CS (**c**), have been used for 100-view imaging. The images are normalized to 1.0. The display window is [0.01 0.55]. Difference images taken from the reference image are shown in the bottom row. The display window is [-0.25 0.25] for the difference images.

We have reconstructed rat abdomen images and pelvic floor images, with the matrix size of 512 × 512 and the pixel size of 120 × 120 μm^2^, using FBP from the 100-, and 900-view projection data. Figure 6 shows the rat abdomen images reconstructed by FBP. Here again, the 900-view FBP image is used as a reference image. The streak artifacts in the rat abdomen images are less conspicuous than they are in the contrast phantom images, but we can see clear streak artifacts from the 100-view images. Figure 7 shows the rat abdomen images reconstructed by the three iterative methods. Here again, the ART image (Figure 7a) shows the strongest streak artifacts and the SAS-CS image (Figure 7c) show less streak artifacts than the CS image (Figure 7b). Figure 8 shows the rat pelvic floor images reconstructed by FBP from the 100- and 900-view projection data. Due to the many bones on the pelvic floor, the 100-view FBP image shows strong streak artifacts. Figure 9 shows the pelvic floor images reconstructed by the iterative methods and the difference images taken from the reference image. Due to the high-intensity bones on the imaging plane, SAS-CS images (Figure 9c) also show residual streak artifacts which are, however, far less than the ART image (Figure 9a) and the CS image (Figure 9b). Table 2 summarizes the RRMEs and SIs of the rat pelvic images reconstructed by the aforementioned three methods with respect to the reference image. Due to the strong streak artifacts, the ART image shows the biggest RRME whilst the SAS-CS image shows the least RRMEs. In terms of the normalized SI, the ART image also shows the worst performance and the SAS-CS image shows the best performance.

**Table 2:** RRMEs and SIs in the rat abdomen images and rat pelvic floor images

**Reconstruction methods**	**Rat abdomen case**		**Rat pelvic floor case**	
	**RRME**	**SI**	**RRME**	**SI**
ART	0.0077	0.4959	0.0108	0.4480
CS	0.0051	0.3790	0.0046	0.2573
SAS-CS	0.0043	0.3567	0.0031	0.2300

Three iterative reconstruction methods, ART, CS, and SAS-CS, are compared for the 100-view imaging.

## Discussion

In sparse-view imaging, reducing the extensive computation time of the CS-based image reconstruction is a great technical challenge for its application to clinical practice [11]. Repetitive forward and backward projections account for most of the computations in the CS-based image reconstruction. Recent innovations in fast iterative image reconstructions based on graphic processing units (GPUs) have shown that sparse-view imaging may gain clinical applications in the near future [29,30]. The computing cost of the CS-based image reconstruction is known to be higher than that of ART, which largely depends on the number of iterations to solve the minimization problem. Therefore, convergence speed of the CS-based image reconstruction is a crucial factor for its use in clinical practice. Recent development of a fast CS-based reconstruction algorithm based on the Barzilai-Borwein formulation has reduced the number of iterations to the extent that the CS-based image reconstruction could be used for real-time IGRT [13].

The computing cost of the proposed method, so called SAS-CS, has been found to be similar to that of the conventional CS-based reconstruction in that SAS-CS needs similar number of iterations. In addition to the repetitive forward and backward projections, SAS-CS needs additional non-iterative computations for the bone component subtraction from the measured sinogram. But, the computing cost for the bone segmentation is minimal as compared to that of the iterative computations. In fact, we have observed that SAS-CS slightly accelerates the convergence of the minimization. It seems that excluding the bone components from the measured projection data in the first iteration (step 5) of SAS-CS accounts for the convergence acceleration. In the second iteration (step 7) in which the bone components are also taken into account, the number of iterations similar to the one in step 5 suffice for further refinement of the reconstructed image in most cases. But we still need to speed up the computation for practical use of the proposed method. Recently developed fast algorithms, such as the adaptive-steepest-descent projection onto convex sets (ASD-POCS) [14,19] or the Barzilai-Borwein formulation-based algorithm [13], may be used for our future studies to reduce the computation time.

## Conclusions

In conclusion, the proposed method can suppress streak artifacts stemming from high-intensity objects in sparse-view CT imaging without significant increase of computing cost as compared to CS- or ART-based reconstructions. Experimental results obtained from the micro-CT imaging of a laboratory rat have demonstrated efficacy of the proposed method in suppressing bone-induced streak artifacts in sparse-view CT imaging.

## Competing interests

The authors declare that they have no competing interests.

## Authors’ contributions

SO and OK conceived the study, implemented the proposed idea, evaluated the reconstructed images, and drafted the manuscript. JG and SY designed and performed the experiments, advised on the image quality evaluation. SY and OK supervised the study. All authors have read and approved the final manuscript.
